# Ocular Blood Flow Autoregulation Mechanisms and Methods

**DOI:** 10.1155/2015/864871

**Published:** 2015-10-21

**Authors:** Xue Luo, Yu-meng Shen, Meng-nan Jiang, Xiang-feng Lou, Yin Shen

**Affiliations:** Eye Center, Renmin Hospital of Wuhan University, Wuhan University, Wuhan, Hubei 430060, China

## Abstract

The main function of ocular blood flow is to supply sufficient oxygen and nutrients to the eye. Local blood vessels resistance regulates overall blood distribution to the eye and can vary rapidly over time depending on ocular need. Under normal conditions, the relation between blood flow and perfusion pressure in the eye is autoregulated. Basically, autoregulation is a capacity to maintain a relatively constant level of blood flow in the presence of changes in ocular perfusion pressure and varied metabolic demand. In addition, ocular blood flow dysregulation has been demonstrated as an independent risk factor to many ocular diseases. For instance, ocular perfusion pressure plays key role in the progression of retinopathy such as glaucoma and diabetic retinopathy. In this review, different direct and indirect techniques to measure ocular blood flow and the effect of myogenic and neurogenic mechanisms on ocular blood flow are discussed. Moreover, ocular blood flow regulation in ocular disease will be described.

## 1. Introduction

Ocular blood flow regulation compensates for changes in ocular activity, keeping the relative constant ocular temperature and retinal perfusion pressure [1]. Recently, there have been dramatic advances in understanding ocular blood flow (OBF) physiology [2]. Autoregulation of blood flow adjustment can be classified into two types static and dynamic according to responding rate [3]. Static autoregulation involves several diverse factors, including myogenic, neurogenic, and metabolic factors [2, 4, 5]; dynamic autoregulation is an instantaneous process facing up sudden variation in perfusion pressure. Dynamic autoregulation of outer ocular vascular system has been extensively studied and revealed a rich sympathetic innervation in the outer ocular vessels [6–8]. In this review, we will review the techniques for ocular blood flow evaluating and focus on the association between autoregulation of blood flow summarizing present knowledge of autoregulatory processes in the regulation of ocular blood flow and its relevance for ocular disease. More importantly, the need for a comprehensive understanding of the mechanisms regulating retinal blood flow is required to gain further insight into the pathophysiology of ocular disease [9].

## 2. Ocular Blood Flow and Anatomy

The retina receives its nutrients from both choroidal and retinal blood flow. Researchers characterize retinal blood flow as a high level of oxygen extraction and a low level of blood flow. The choroidal vascular beds supply nutrition to the optic nerve [10]. The interplay among them may be essential for maintaining a healthy optic nerve [4].

The physiology and anatomy of the retinal circulation appear like the brain circulation; meanwhile the retinal circulation does not have autonomic innervation. The presence of endothelial tight junctions results in a blood-retinal barrier, resembling the blood-brain barrier. Large studies indicate that autoregulation may be less effective to the retina but better to the choroid [11–14]. Optimal visual function needs an appropriately regulated environment. As reported, epithelia and vascular endothelium as dynamic structures identify this regulation. These structures quickly respond to changing physiological needs and extrinsic conditions.

Many studies have demonstrated that efficient autoregulation of ocular blood flow in the ocular nerve head (ONH) is potential taken by increasing capacitance of blood vessels. The changing magnitude of the reactive increased vascular capacitance compensates the decrease of ocular nerve head vascular resistance with intraocular pressure increasing [13].

## 3. Techniques for Ocular Blood Flow Evaluating

As large methods have been described in previous research, no single vascular indicator can completely evaluate ocular blood flow [15, 16]. Every technique measures its specific aspects of ocular circulation, each with different limitations but providing a view of ocular hemodynamics [14]: pulsatile ocular blood flow, a possible indication of choroidal blood flow [17, 18]; color Doppler imaging (CDI), a widely used assessment to evaluate retrobulbar vascular circulation [19]; scanning laser Doppler flowmeter, for quantifying superficial layers of ONH and retinal vascular circulation [19, 20]; and optical coherence tomography (OCT), for detecting noninvasive vascular mapping at the microcirculation level.

### 3.1. Color Doppler Imaging

CDI has been widely used to investigate retrobulbar vascular parameters including blood velocity, pulsatility index, and resistive index, in both health and disease [9]. But CDI technology has its own major limitation. CDI quantifies the vascular velocity rather than vessel diameter [21]. However, the consistent correlation between blood flow and vascular velocity has been identified; moreover, the measurements of blood flow are viewed to be reproducible [19, 22]. CDI may be particularly useful in cases with media opacities.

### 3.2. Doppler Fourier Domain Optical Coherence Tomography (Doppler FD-OCT)

One of the main advantages of the technique over the existing methods of measuring retinal blood flow is its ability to rapidly provide the total retinal blood flow (TRBF) by summing all measures around the optic nerve head, thereby assessing the whole retinal blood flow rather than a single point within the retinal vascular tree [23]. There are also still some limitations to the double circular scanning method that need to be addressed in the future development of the technique. These include complete elimination of eye motion, which can resolve possible errors in Doppler angle measurement, and full automation of the software for objective and reliable delineation and detection of vessel area [9].

### 3.3. Angiography

Fluorescein angiography is the gold standard for* in vivo* evaluation of retinal circulation. It provides useful qualitative information [24]; however, it has its advantage on investigating the superficial ocular nerve head vascular and its limitation on deep ocular nerve head circulation [25]. Although the passage time of fluorescent dyes through ocular vessels may not be highly correlated with OBF, it gives useful information on ocular perfusion [26].

### 3.4. Split-Spectrum Amplitude-Decorrelation Angiography- (SSADA-) OCT

Very recently, using ultrahigh-speed OCT, researchers developed a new method using 3D angiography algorithm to image ophthalmic microcirculation, which is called split-spectrum amplitude-decorrelation angiography (SSADA) [27]. OCT angiography generated by the latest SSADA measures optic disc perfusion and may be helpful in the evaluation of OBF [28]. The major limitation associated with OCT angiography is that SSADA-OCT only yields a flow index in arbitrary units instead of absolute volumetric flow. Although various methods can be capable of evaluating ophthalmic hemodynamics, like Heidelberg Retinal Flowmeter (HRF) and Laser Doppler Flowmetry (LDF), the direct evaluation of microcirculation is the most promising method in need [29].

## 4. Mechanism and Modulation of Ocular Blood Flow Regulation

Ocular blood flow autoregulation is known to fit well with changes in OPP, assembling other human organs and tissues. Autoregulation keeps blood flow relatively constant, only increasing blow flow in response to metabolic demands in the eye. However, defective autoregulation may exert its important role in the pathophysiology of ophthalmic vascular diseases [30].

The common method that clinicians use to assess autoregulation ability is artificially elevating or decreasing OPP in accordance with different results of blood flow evaluation. The classic curve of autoregulation describes the relation that the blood flow changes are followed by OPP changes within a certain range [30, 31]. In contrast to the clinic, autoregulation capacity in the eyes of both experimental animals and humans is measured by a “two-point” BF assessment. If blood flow deviates dramatically in response to pressure changes, therefore we can consider autoregulation impaired [32–37].

As discussed above, autoregulation is classified into two types to adjust blood flow changes, namely, static and dynamic. During and after a perturbation, static autoregulation system requires several minutes to induce a new steady balance of blood flow; on the other hand, the dynamic autoregulatory responses occur within 5 s [2, 5]. Various factors, including metabolic, myogenic, and neurogenic factors, are involved in static autoregulation within longer time to regulate the blood flow. On the other hand, factors that are involved in dynamic autoregulation, an instantaneous reaction to sudden changes in perfusion pressure, are found to be quite contractive [38].

Different from choroidal circulation, there is no neuronal innervation in retinal vascular beds. Retinal and ONH blood flow are mainly in the regulation of local mechanisms. Beside myogenic factor, endothelial cells take part in local regulation [39–43]. The mediators of these mechanisms include oxygen, carbon dioxide, angiotensin-II, adenosine, nitric oxide (NO), and endothelin-1 [44]. Among these factors, the role of angiotensin-II and endothelin-1 in regulating the blood flow of retina and ONH is disputable. Due to exclusion by the blood-retinal barrier, circulating hormones like angiotensin-II and endothelin-1 cannot directly be transported to smooth-muscle cells and therefore do not participate in retinal blood flow regulation in healthy persons. But they could diffuse from choroid into retinal tissue in the incomplete blood-retina barrier.

However, sympathetic innervation plays an important role in choroidal blood flow regulation. Recent research revealed that the choroid mainly controlled by the sympathetic nervous system and metabolic factors [45, 46] can be autoregulated in response to an increase or decrease in OPP.

### 4.1. Endothelin-1 (ET-1)

Endothelin-1 is mostly secreted by the endothelial cell, as the potent vasoconstrictor has been found to affect vascular endothelium and pericyte interactions within the ophthalmologic microcirculation [47]. As reported, in healthy humans, ET-1 affects the regulation of ophthalmologic posterior parts, especially choroidal blood flow regulation [48]. ET-1 has two types of binding sites, ET_A_ receptor and ET_B_ receptor, reducing ocular blood flow and mediating preferably binds to the ET_A_ receptor expressed on vascular smooth-muscle cells mediating vasodilatation by releasing prostacyclin and NO, respectively. Increased ET-1 concentration has been observed in the aqueous humor of glaucoma patients [49]. Moreover, the increased ET-1 levels elevate IOP, inducing decreased ocular blood flow and astrocyte proliferation and therefore may cause the degeneration of retinal ganglion cell in the end [50]. Thus, ET-1, as a major risk factor, exerts function in the process of retinal disease, such as glaucoma and diabetic retinopathy. Because of ET receptor antagonist reducing the retinal blood flow, endothelin antagonism is considered as a promising therapy for glaucoma [51]. In recent decades, calcium channel blockers (CCBs) have been commonly available to improve BF regulation and reduce the vasoconstrictive effect of endothelin-1 [52]. The effect of CCB on OBF has been investigated in numerous studies. But not all studies investigating CCBs indicate an impact on ocular blood flow.

### 4.2. Nitric Oxide

Nitric oxide (NO) plays a potential role in vascular diastole and protecting vascular endothelial cells from the risk of glaucoma and diabetic retinopathy [53, 54]. Nitric oxide synthase (NOS) is divided into three isoforms to perform dichotomous function, including endothelial nitric oxide synthase (eNOS), neural nitric oxide synthase (nNOS), and inducible nitric oxide synthase (iNOS). Only in allergic or inflammatory conditions is iNOS expressed, while eNOS and nNOS are constitutively expressed in the retina and the choroid. NOS influences both pathological and physiological processes of the eye and regulates ocular blood flow and intraocular pressure (IOP) [54]. Impaired NOS signaling is induced by vascular dysfunction in POAG and diabetic retinopathy [55].

### 4.3. Estrogen

Estrogens appear to be neuroprotective and also have been shown to improve retrobulbar circulation; recent research indicates significantly improved retinal blood flow and reduced risk for developing glaucoma in postmenopausal women with hormone replacement therapy [44, 56].

### 4.4. Diurnal Variations

Recently, there is evidence supporting the function of diurnal rhythm in the ophthalmologic blood flow regulation. Compared with retinal and ONH blood flow, diurnal variation of choroidal circulation was more easily affected by systemic environment changes [57]. To our knowledge, the relation between IOP and diurnal variations has been investigated for a long time.

### 4.5. Adenosine

In healthy humans, there is sufficient evidence that adenosine has been demonstrated to have effect on retinal vasodilatation and modulating IOP [58]. Acting through adenosine A1, A2, and A3 receptors, adenosine has been proposed for stimulating adenylyl cyclase and subsequently regulating the activation of different ion channels such as reducing calcium influx or activating chloride channel [59]. Intravenous administration of adenosine analogs was applied in clinical trials to regulate blood flow [60].

### 4.6. Carbonic Anhydrase

Carbonic anhydrase especially CAII has been considered as an important factor in retinopathy. Inhibition of this enzyme is thought to reduce IOP by increasing aqueous outflow. Carbonic anhydrase inhibitors (CAIs) have been shown to have a positive effect on ocular blood flow and are utilized to reduce IOP in patients with OAG [61].

### 4.7. Myogenic Mechanisms

There is disputable evidence on myogenic autoregulation. Most studies predict that myogenic mechanisms do not have significant effect on ocular blood flow autoregulation. Stretching of the vessel wall leads to activating calcium channels resulting in an increase of calcium influx and vascular constriction. With elevating perfusion pressure, inherent responsive vasoconstriction is thought to rapidly adapt to blood flow changes [62].

## 5. Impaired Autoregulation of Ocular Blood Flow in Ocular Diseases

### 5.1. Open-Angle Glaucoma

Glaucoma is a progressive optic nerve disease with characteristic changes in the structure of the optic nerve and vision loss. Vascular regulatory plays an important role in the progression of retinopathy such as OAG and DR [63]. There is sufficient evidence indicating a correlation of blood pressure with either ocular vascular diameter or blood velocity in patients with ocular hypertension and OAG, rather than in health, demonstrating impaired autoregulation in OAG [64]. But autoregulation of ocular blood flow is difficult to assess, and clear criteria to classify the status of autoregulation as dysfunctional or functional remain scarce. On the other hand, the majority of studies have demonstrated that individuals with progressive glaucoma have lower blood flow parameters than individuals with stable vision loss; therefore the reduced blood velocity is a risk factor for the progression of OAG [65]. In particular, the disturbed perfusion system, or vascular dysregulation to compensate the blood flow requirement or to fluctuate in perfusion pressure, may result in chronic changes or low ocular perfusion. Many researchers discover the signs of reduced ocular perfusion in the early and advanced diagnosis of OAG. Fluctuant ocular perfusion, in turn, may cause oxidative stress reaction and ischemic damage, potentially resulting in glaucomatous damage at ONH.

There is evidence indicating that autoregulation is disturbed in glaucoma, resulting in the retinal vascular parameters response to OPP changes to be more passive, elevating to the higher level when the OPP rises or reducing to the lower level if the OPP drops [8, 52, 66–70]. By using CDI, OAG is characterized by damage of retinal nerve fiber layer (RNFL) reduction of vascular circulation and changes of blood rheology [71], while there is debatable evidence that impaired autoregulation of ocular nerve head blood flow has been found in patients with OAG [37, 72].

A lack of autoregulation, a vasospastic reaction to stimuli such as psychological stress or cold, has been considered as a possible contributing factor to OAG [73], particularly without associated IOP [74]. Moreover, the variation range of intraocular pressure before treatment is crucial in observing how effectively this vascular factor is regulated in glaucoma patients [75]. The mentioned therapies in the review, including *β*-blockers and carbonic anhydrase inhibitor, are most commonly used to lower IOP and subsequently prevent optic nerve damage and progression of the disease.

### 5.2. Diabetic Retinopathy

Retinopathy is a common complication of diabetes mellitus and one of the leading causes of irreversible blindness in the developed world. In the early stage of diabetic, retinal integral network could successfully adapt to the systemic metabolic changes. But when it progresses, oxygen and nutrient requirements of the retina may lead to eventual loss of ocular homeostasis. The specific characteristic structural changes of DR have focused on damage to the retinal vessels, no matter in nonproliferative diabetic retinopathy (NPDR) or proliferative diabetic retinopathy (PDR) [76]. All vascular lesions of DR include the appearance of microaneurysms, vascular nonperfusion, degeneration, and neovascular stage. However, the effect of ocular blood flow regulation on DR remains unclear. There are several disputable views of dysfunctional flood flow in the orbital vascular in diabetic retinopathy patients. To investigate the blood flow of DR, different vascular parameters were observed in recent studies [77]. As reported, compared with normal individuals, DR patients had a significantly higher resistivity index in the ophthalmic artery and central retinal vein and lower PSV and EDV of the posterior vascular [78]. However, there is conflicting evidence that different blood flow velocity changes were not consistent in different techniques and measurements. Recently antivascular endothelial growth factor (VEGF) is the most popular therapy to interfere with autoregulation of the choroidal and retinal microcirculation, resulting in reducing the progression of neovascularization and the incidence of blindness in patients with DR [79].

## Perspective

In future studies, it is critical to develop the measurement of blood flow regulation and further study the effect of ocular blood flow regulation on the incidence and progression of glaucoma and diabetic retinopathy. If relevance to this retinopathy risk or development can be established, the clinical implications for patient management should be considered, particularly for individuals with disturbed ocular vascular circulation [80]. Looking forward, more study of OBF needs to be focused on in the future. The development and up-to-state technology to measure OBF are more precise; we also need more direct data on blood flow in specific tissues of the eye [14].
